# Computational models for generating microvascular structures: Investigations beyond medical imaging resolution

**DOI:** 10.1002/wsbm.1579

**Published:** 2022-07-26

**Authors:** Cameron Apeldoorn, Soroush Safaei, Julian Paton, Gonzalo D. Maso Talou

**Affiliations:** ^1^ Auckland Bioengineering Institute, The University of Auckland Auckland New Zealand; ^2^ Cardiovascular Autonomic Research Cluster, Department of Physiology, Faculty of Medical and Health Sciences The University of Auckland Auckland New Zealand

**Keywords:** angiogenesis, arteriogenesis, cancer, computational model, pruning

## Abstract

Angiogenesis, arteriogenesis, and pruning are revascularization processes essential to our natural vascular development and adaptation, as well as central players in the onset and development of pathologies such as tumoral growth and stroke recovery. Computational modeling allows for repeatable experimentation and exploration of these complex biological processes. In this review, we provide an introduction to the biological understanding of the vascular adaptation processes of sprouting angiogenesis, intussusceptive angiogenesis, anastomosis, pruning, and arteriogenesis, discussing some of the more significant contributions made to the computational modeling of these processes. Each computational model represents a theoretical framework for how biology functions, and with rises in computing power and study of the problem these frameworks become more accurate and complete. We highlight physiological, pathological, and technological applications that can be benefit from the advances performed by these models, and we also identify which elements of the biology are underexplored in the current state‐of‐the‐art computational models.

This article is categorized under:

Cancer > Computational Models
Cardiovascular Diseases > Computational Models

## Graphical abstract

Abstraction of the formation of new vascular connections through angiogenesis.

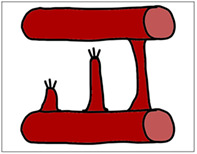

## 1 INTRODUCTION

The vasculature is the core transport system of a large number of species in this planet. It extends throughout the entire body of a specimen and ranges in scale from large conduit arteries and great veins to microvascular components such as arterioles, capillaries, and venules. These components are integrated in a complex dynamic system of networks, providing the means to transport oxygen and nutrients to cells throughout the organism. Due to the large heterogeneity in tissue composition of the biological system and changes in cellular metabolic demands, development and adaptation of these vast networks is of utmost importance for maintenance and functional adaptation of tissues in the organism.

With advances in imaging, mathematical techniques, and computational resources, we have become capable of mathematically modeling the processes of development and adaptation of these microvascular components when metabolic demands of the surrounding tissue are altered or when new tissue growths (e.g., wound healing, tumoral development). In this article, we review different approaches to modeling the growth, adaptation and pruning behaviors of capillaries at the lowest level of the vascular network. We place a special focus on the usability of these in silico methods from the perspective that they will provide unique mechanistic insights, as well as making valuable predictions in both in vivo and in vitro settings.

### 1.1 Scope

This work aims to describe the state‐of‐the‐art in the mathematical modeling of microvascular development and adaptation, involving angiogenesis, arteriogenesis, and pruning processes. Early embryological processes involved in the formation of the cardiovascular system such as vasculogenesis are out of the scope of this review. In terms of organization levels of biological systems, we focus on works modeling the processes at tissue, cellular, and molecular levels, with special focus on the tissue level as it allows us to understand the interactions underlying development and adaptation. We excluded works at the organ level so we can focus on general processes of angiogenesis and general purpose models. However, we discuss the application of the models to specific pathologies of biological processes in Section 4. In terms of scales, we restrict the review to models working on arterioles, venules, and capillaries. Lastly, we have excluded pathological angiogenic processes—for example, vascular mimicry and blood vessel cooption (Carmeliet & Jain, 2011; Hillen & Griffioen, 2007)—from the biological background and model analyses, as these processes are exclusive to tumoral growth rather than being a pathological expression of general physiologic angiogenesis processes.

### 1.2 Manuscript structure

The manuscript is structured in three main sections: biological background, computational models, and applications. In the biological background, we present the current understanding of the microvascular processes of development and adaptation, as well as, the driving forces involved in the execution and initiation of the processes. In the computational models section, we present the works in the literature that aim to model such microvascular processes, assessing which of the biological components have been modeled and at which organizational level. In the applications section, we discuss the opportunities for future translation of these models. Finally, discussions and conclusions of the review are outlined, highlighting strengths of the current models and gaps in the literature for future development.

## 2 BIOLOGICAL BACKGROUND

The formation of new vascular networks, their continued development and remodeling are components of a multiscale process driven by local metabolic demand and systemic homeostatic principles. At the cellular level, endothelial cells multiply, migrate, aggregate, and organize themselves in response to stimuli provided by their local conditions and signaling from nearby cells. The actions of endothelial cells make up the core of the underlying physiological processes that form and adapt vascular networks.

Body cells consume oxygen and various nutrients to satisfy their metabolic demands. These resources are supplied through the vascular network. When cells receive insufficient resources, they release signaling molecules that in turn trigger *physiological processes of vascular adaptation*. The vascular network adapts to satisfy the resource consumption of the cells while also sustaining itself and satisfying the general drive to efficiency in terms of energy consumption. These goals manifest as a pair of competing processes: vessel generation/adaptation versus vessel removal. The competition of these processes results in a homeostatic state matching delivery of resources to tissue demand while removing unnecessary vessels.

### 2.1 Physiological processes of vascular adaptation

The remodeling of the vasculature in normal development is driven by three different processes: (i) angiogenesis; (ii) arteriogenesis; and (iii) pruning. Equally important is the tissue being vascularized, as that defines the consumption of oxygen and nutrients, triggering the adaptation of the vascular network.

**FIGURE 1:**
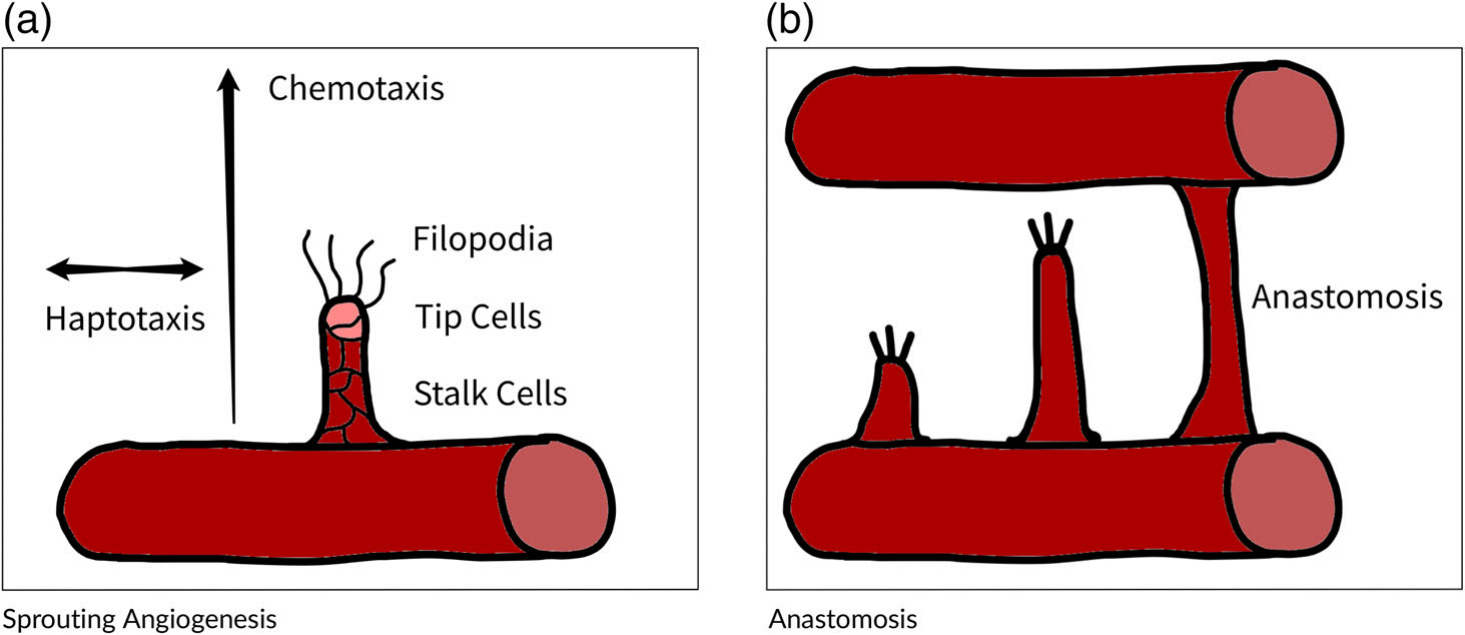
Angiogenic growth processes: new vessels start to grow in the existing vasculature, typically initiated by vascular endothelial growth factor (VEGF) as a reaction to hypoxia (low oxygen levels) in the tissue

Angiogenesis is the process by which new vessels start to grow in the existing vasculature (Buschmann & Schaper, 1999; Heil et al., 2006; see Figure 1). It is typically initiated by vascular endothelial growth factor (VEGF) as a reaction to hypoxia (low oxygen levels) in the tissue (Heil et al., 2006). In turn, there are two different angiogenic processes: sprouting angiogenesis (SA) and intussusceptive angiogenesis (IA). In SA, new vessels sprout from the existing vasculature to reach the hypoxic zone where they connect (anastomose) with other sprouting or existing vessels to form loops through which blood can flow and deliver resources (Betz et al., 2016; Chaplain, 2000; De Spiegelaere et al., 2012; Eilken & Adams, 2010). In IA, a capillary is split into two new capillaries via a process involving the proliferation of endothelial cells and a separation process driven by pericytes (De Spiegelaere et al., 2012). Depending on the resultant branching of the generated vessels, we can differentiate between three types of IA: intussusceptive microvascular growth, intussusceptive arborization, and intussusceptive branching remodeling (De Spiegelaere et al., 2012; Djonov et al., 2003; Kurz et al., 2003).

**FIGURE 2:**
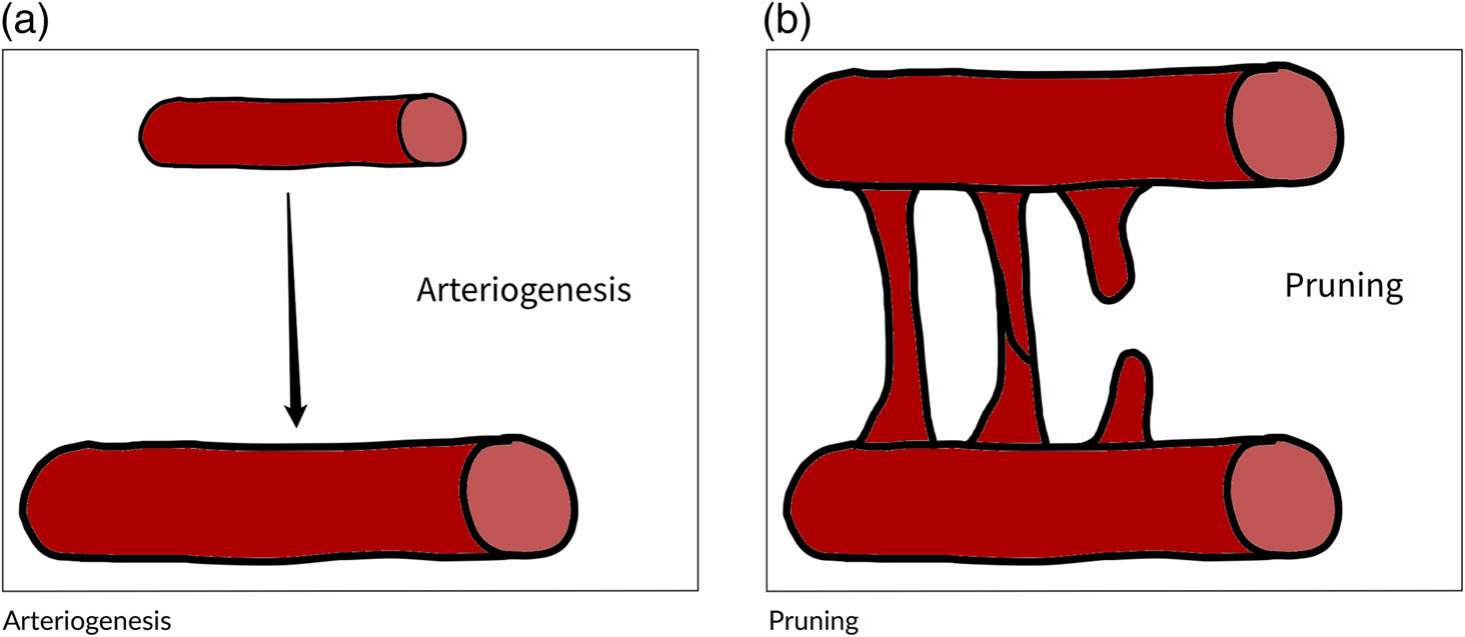
Adaptation and maintenance processes. (a) Arteriogenesis: vessels increase their diameter and the number of smooth muscle cells in vessel wall driven by internal mechanical stresses. (b) Pruning: reabsorption of vasculature in response to blood flow reduction

Arteriogenesis is the process by which existing vessels increase their diameter via endothelial and subsequent smooth muscle proliferation (Schaper & Buschmann, 1999; see Figure 2a). Different from angiogenesis, arteriogenesis is not triggered by local hypoxia, although it may serve alleviate hypoxia of downstream perfused tissues by increasing local blood flow. It is induced by mechanical forces such as increased wall shear stress in the endothelial cells (Buschmann & Schaper, 1999; Heil et al., 2006). An example of this is the remodeling of the collateral vessels (small vessels that perfuse the same tissue from different vessels) of an artery with stenosis. As the stenosis increases, the vessel's distal pressure is reduced. Consequently, collateral vessels have a larger pressure gradient and experience increased blood flow and wall shear stress promoting arteriogenesis.

Finally, pruning is the process of reabsorption of low‐pressure gradient vessels which, as a consequence, exhibit a low wall shear stress (Chen et al., 2012; see Figure 2b). As low blood pressure gradients promote the formation of blood clots due to blood stagnation, this process has an important role in the maintenance of the vascular bed. Additionally there is evidence that the pruning process acts to form a homeostatic balance with the growth processes (Pries & Secomb, 2014).

#### 2.1.1 Sprouting angiogenesis

SA is the process by which new vessels grow out of the existing vasculature in response to signaling growth factors like VEGF. It is the primary means of growing vessels in adulthood. When a cell triggers SA, nearby endothelial cells will migrate and begin to multiply forming a sprout, which can be divided into tip cells and stalk cells. The tip cells move through the surrounding extracellular matrix in the direction of the gradient of growth factors (Gerhardt et al., 2003), and the stalk cells proliferate to maintain a continuous growing vessel segment between the tip and the original vessel called a sprout.

In normal physiological conditions, the growth factor driving the sprouting process will be VEGF released by hypoxic cells lacking sufficient oxygen diffused from the surrounding vasculature. However, considerable study (Ellis & Hicklin, 2008; Hicklin & Ellis, 2005; Kerbel, 2008; Weis & Cheresh, 2011) has been devoted to the pathological case of a tumor in which there is an excess of angiogenic growth factors released due to the presence of hypoxic tissue within the tumor growth.

When an event induces SA, there is a period of 1–2 days before the sprouting begins in earnest. The sprouting continues until the conditions causing VEGF expression are alleviated by the aggregation of new viable vessels, which will take somewhere from 3 days to a couple of weeks (Baker et al., 2012; Folkman, 1984; Kenyon et al., 1996).

Vessels are formed from interconnected endothelial cells arranged in a tube formation. These endothelial cells express receptor tyrosine kinase (RTK) on their surfaces which binds and is signaled by angiogenic factors (Olsson et al., 2006; Tammela et al., 2008), which trigger the angiogenic process. There are a variety of RTK pathways for different angiogenic factors and the precise nature of their expression is beyond the scope of this article (Covassin et al., 2006; Olsson et al., 2006), however, the outcome of these pathways is that some endothelial cells will develop into tip cells. These tip cells will locally inhibit the expression of other tip cells through the Delta‐Notch signaling pathway to maintain local vessel network integrity (Hellström et al., 2007; Lobov et al., 2007), develop filopodia which sense the direction of the gradient of the angiogenic factor, and allow the tip cells to migrate along the concentration gradient in a process called chemotaxis (Gerhardt et al., 2003). As the cells navigate the extracellular matrix they experience haptotatic forces which generate movement unaligned with the primary chemical gradient.

As tip cells migrate, stalk cells proliferate to maintain the connection between the tip cell and the original vessel. Stalk cells have their own pro‐angiogenic regulator called Jagged1, which is antagonistic to the pathways that cause the development of tip cells (Benedito et al., 2009). Stalk cells are relatively static compared to tip cells and appear in the path followed by the tip cells in what is called the snail‐trail effect.

#### 2.1.2 Anastomosis

Anastomosis is the process by which tip cells connect to other vessel segments or tip cells to form complete loops as part of a vascular network. As part of this process, the tip cell fuses with the target vessel/tip cell by interconnecting its proximal lumen (constituted by stalk cells) with the lumen on the other end, forming an complete endothelial tubule allowing for fluid flow through the combined structure. The established blood flow promotes vessel stabilization and pericytes recruitment leading to a new functional vessel (Armulik et al., 2011; Bouïs et al., 2006).

Tip cell production and movement is primarily driven by chemotactic and haptotactic forces, however, in the final stages before anastomosis, additional elements serve to guide and promote the ending of the sprouting process. In particular, macrophages have been found to chaperone tip cells to anastomosis (Fantin et al., 2010) and the filopodia on the tip cells are capable of sensing nearby sprouts and directing migration to form complete vessel segments (Phng et al., 2013).

After an anastomosis has occurred, the blood flow distribution will be adjusted to account for the new vasculature. This occurs over a period of minutes, a faster timescale compared to the sprouting and pruning processes (Chaplain et al., 2006).

Anastomosis is important from a modeling perspective as it corresponds to the end of the sprouting growth process and the start of fluid flow through a segment making it a computationally relevant breakpoint for the transition from growth to resource transport. Anastomosis also represents the beginning of the transition of tip and stalk cells back to regular endothelial cells as their function is complete.

#### 2.1.3 Intussusceptive angiogenesis

IA is the process by which new blood vessels are generated by the splitting of existing blood vessels. In the IA process, endothelial cells of opposite sides of the vessel advance toward the lumen building a double‐layer intraluminar tissue pillar. Interendothelial cell junctions are remodeled and layers of endothelial cells in the pillar detach from each other, splitting the vessel into two. Finally, migration of pericytes and myofibroblasts stabilize the newly generated vessel surfaces by generating the extracellular matrix (Burri & Djonov, 2002; De Spiegelaere et al., 2012). Via this mechanism, IA participates in the genesis and refinement of vascular plexuses (intussusceptive arborization), remodeling of vascular junctions (intussusceptive branching remodeling) and, even, execution of complex pruning processes (intussusceptive pruning; Burri & Djonov, 2002; De Spiegelaere et al., 2012).

Due to the reuse of existent endothelial cells, IA allows for rapid expansion and remodeling of the vascular network at low metabolic cost due to the lack of cell proliferation (Djonov et al., 2002). The intussusceptive process is desirable in scenarios requiring an increase in effective surface area of the vascular network—increasing the local diffusion of oxygen into the tissue—such as during the growth phase and in response to pathologies such as wound healing. Evidence suggests that while SA and vasculogenesis form the primordial vascular connections in early stages of angiogenesis, IA predominates in the later stages of vascular growth and remodeling (Djonov et al., 2001; Macchiarelli et al., 2006; Makanya et al., 2005). To the best of our knowledge, there is no known clear signaling pathway or triggering process for IA reported in the literature, but it does perform a unique role in network adaptation and as such has been included here for completeness.

#### 2.1.4 Arteriogenesis

Arteriogenesis is an adaptive process through which vessels increase their diameter and the number of smooth muscle cells in vessel wall. Unlike the sprouting process, arteriogenesis is not driven by local hypoxic conditions but rather by internal mechanical stresses (Schaper & Scholz, 2003), primarily via wall shear stress (Cai & Schaper, 2008; Helisch & Schaper, 2003). Increasing the diameter of a vessel decreases its pressure drop and alleviates wall shear stresses. By means of this mechanism, arteriogenesis is a key contributor to the development of collateral arteries which enable continued perfusion in the event of occlusion of major vessels (Grundmann et al., 2007; Hoefer et al., 2006; Seiler et al., 2013).

The exact sensing pathway connecting the mechanical stress stimuli to the arteriogenesis process remains unclear (Deindl & Schaper, 2005). However, there is evidence that endothelial cells play a key role in sensing changes in flow and pressure derived stresses, particularly, the wall shear stress (Ma & Bai, 2020).

Increasing local oxygen diffusion is the side effect of the distal increment in blood flow, consequence of arteriogenesis. As such, it can affect distal hypoxic cells, however, as previously stated, resolving hypoxia is not a primary driver or effect of the arteriogenesis process.

#### 2.1.5 Pruning

Pruning is a process, which occurs at every scale of the vascular network and is a remodeling behavior that contributes to reduce the energy required for tissue perfusion. Whereas sprouting occurs as a response to cellular needs, pruning may derive as an evolutionary response to reduce the effort needed by the cardiac muscle to perfuse the tissues with blood. This process reduces energy expenditure by optimizing the existing vessel network to minimize the total vessel length and surface while still maintaining the required function.

As new vessels grow as part of the sprouting process or expand through arteriogenesis the most efficient arrangement of the vessels will shift. The pruning process reduces the inefficiency of the current vasculature in response to this shift. As a part of this process, vessel connections will migrate, vessel segments will shift, and superfluous elements of the network can be reabsorbed through apoptosis and macrophage consumption (Poché et al., 2015).

One of the better characterized processes to observe the pruning mechanism is the transformation that occurs at the end of retinal development. During retinal development, a vascular network is needed to provide resources for retinal construction. However, proper retinal function requires the free passage of light and, as such, a vessel regression event occurs to remove the retinal vasculature through the use of pruning (Fruttiger, 2007). Unfortunately, major organ specific vessel regression events, such as in the retina or in the mammary glands post‐lactation, do not follow the same processes as the continuous pruning behaviors that occur in the general vascular remodeling task (Korn & Augustin, 2015).

In general developmental remodeling, the pruning process begins with endothelial cells constricting the vessel leading to reduced flow and then static flow via vessel occlusion. The endothelial cells affected by the static flow undergo a mixture of apoptosis and migration, leading the occlusion to become a break and forming two separate vessel segments (Franco et al., 2015; Korn & Augustin, 2015).

The pruning and maintenance process begin to develop 1–2 days after an event occurs to stimulate them (e.g., blood flow insufficiency). This means that during growth, pruning will begin to mitigate the sprouting process after an initial period of uncontested growth (Baker et al., 2012; Folkman, 1984; Kenyon et al., 1996).

The process by which vessel segments are selected for pruning is unclear, however, we do know that blood flow and hemodynamic behavior are major regulators for pruning, with non‐perfused vessels demonstrating a predisposition to pruning (Ando & Yamamoto, 2009; Chen et al., 2012). Furthermore, evidence shows that vessels which suffer temporary obstruction have a predisposition to pruning within 3 weeks even if flow through the vessel resumes (Reeson et al., 2018).

### 2.2 Driving factors of vascular adaptation

The above biological processes are driven by physical requirements and signaling mechanisms. In a physiologically relevant computational model, these components make up the observable inputs to the system that determine its development and response. In this section, we detail the more relevant driving factors related to the previously mentioned processes of vascular adaptation.

#### 2.2.1 Oxygenation

Cells consume energy to drive their function and survival. The aerobic energy system is more effective than the anaerobic energy system. As such, it is physiologically desirable for cells to have enough access to oxygen as it is metabolically required. In the body, oxygen is inspired through the lungs where it binds to hemoglobin in erythrocytes. These blood cells are then transported through the cardiovascular network and oxygen is released to cells in the proximity of vascular segments.

In response to hypoxic conditions, cells generate hypoxia‐inducible factor 1, which greatly promotes the expression of growth factors, particularly VEGF, to signal the need for adaptation of the vascular network to satisfy local oxygenation needs (Forsythe et al., 1996). In addition to the hypoxic response, hyperoxia has been shown to contribute to vessel regression behavior by way of inhibition of the VEGF pathway (Baffert et al., 2006; Claxton & Fruttiger, 2003).

#### 2.2.2 Metabolic requirements

As the primary chemical transport system, the cardiovascular system also provides for other metabolic requirements such as removal of built‐up CO_2_ and provision of glucose to enable aerobic energy processes. Although not as dominant in the signaling of vascular development, other metabolic requirements have been shown to impact the expression of growth factors. In particular, pH and glucose levels have been found to modulate the hypoxic response of retinal Müller cells, but do not induce growth factor expression independently (Brooks et al., 1998). These metabolic requirements lack a clear dominant signaling pathway, unlike oxygenation, but do effect the sensitivity and degree of the hypoxia response pathway (Fantin et al., 2010).

Additionally, angiogenesis is a metabolically expensive process where endothelial cells proliferate to form new vessels. This proliferation is sustained through the consumption of metabolites as energy, generating additional biomass. The pathways that control this shift in metabolism are closely related to those inducing angiogenesis (Potente & Carmeliet, 2017).

#### 2.2.3 Growth factors

Growth factors are the chemicals released by cells, which induce the expansion of the vascular network to fulfill their needs. The primary growth factors involved in angiogenesis are the VEGF sub‐family, although there are more specific angiogenic factors, such as placental growth factor, that may also intervene in different tissues and organs.

VEGF‐A has been shown to trigger and direct the sprouting process. With the concentration of this chemical inducing proliferation and the gradient driving the direction of tip cells migration (Gerhardt et al., 2003). VEGF‐C has also been shown to contribute to vessel growth in retinas, tumors, and in zebrafish embryos (Covassin et al., 2006).

In addition to promoting vessel growth, a certain level of VEGF is required in transient development vessels to prevent regression (Claxton & Fruttiger, 2003). This dependence fades in most adult blood vessels as the major networks become stable (Baffert et al., 2006).

#### 2.2.4 Inhibitors

Accompanying growth factors, there is also a need for inhibitory components to regulate the growth process. Of particular note in SA is the local inhibitory effect of a developing tip cell. When the growth factor concentration becomes high enough to trigger the transition of an endothelial cell into a tip cell, that tip cell inhibits a local region through the Delta‐Notch pathway, where the expression of the protein Delta‐like ligand 4 (Dll4) interacts with the notch ligand in nearby cells to downregulate VEGF receptor proteins to prevent the simultaneous development of multiple tip cells in the same region from destabilizing the existing vasculature (Covassin et al., 2006; Hellström et al., 2007).

Another inhibitory example is in the corneal development process. At first, the cells in the cornea require a vascular network for its early development. However, corneal function requires unblocked light access and as such the final stages of development require the inhibition of growth and resorption of the early vasculature (Ito & Yoshioka, 1999).

#### 2.2.5 Mechanical forces

The vascular adaption is orchestrated through the sensing of hemodynamic and tissue forces. As blood flows, it exerts sheer forces on the vessel wall and as a sprout grows it changes the stress distribution within the host tissue. These varied mechanical forces are generated by and contribute to the regulation of angiogenesis, arteriogenesis, and pruning. Key mechanical elements in these processes are wall shear stress (Schaper & Scholz, 2003) and blood flow (Chen et al., 2012). To facilitate oxygen diffusion, capillaries are thin walled and have a limited capacity to handle wall shear stress. The presence of high wall shear stress may compromise the integrity of the vessel, however the vessel adapts via the arteriogenesis process which enlarges vessels and avoid vascular disruption (Helisch & Schaper, 2003).

It is also necessary for capillaries to maintain a certain level of flow through a vessel as otherwise the blood may become stagnant and deoxygenated causing a failure of function and potential blood clotting. As such, shear sensitive receptors detecting blood flow can trigger the pruning process to resorb vessel segments with insufficient flow (Ando & Yamamoto, 2009).

During the sprouting process of angiogenesis the tip cells migrate through an extracellular space, this movement involves force generated by the cell promoting movement and reaction forces from the extracellular matrix. The balance of these forces defines the resulting movement (Shiu et al., 2005).

## 3 COMPUTATIONAL MODELS

In the previous section, we presented the biological processes and driving factors involved in the development and adaptation of microvascular networks. Based on that knowledge, we perform a comparative and critical analysis of different computational models that paved the way for microvascular modeling. The analysis considers which biological processes and drivers have been accounted for. We acknowledge that not all processes may be relevant for the particular applications addressed in the analyzed works, and evidently their authors decided to remove nonrelevant modeling components.

### 3.1 Tissue level models

Angiogenesis is a problem that operates on multiple organization levels. Balding and McElwain (1985) established what has come to be the foundation of the tissue level modeling of angiogenesis. Based on previous works on fungal growth modeling, they proposed a series of partial differential equations (PDE) to track the concentration of key elements (tip cell density, capillary density, and tumor angiogenic factor [TAF] concentration) involved in the angiogenesis process as a response to tumoral growth.

This tissue level approach works by tracking key elements and defines a series of rules defining their interactions. In Balding and McElwain's initial model capillary density is assumed to increase through the movement of tip cells and decrease through a decay process that approximates pruning. Tip cell density is increased by a linear function of TAF concentration and capillary density, and decreased by a function of tip cell density and capillary density. Tip cells movement consists of chemotactic flux along the gradient of the chemo attractant TAF and random movement. TAF concentrations were determined from initial conditions by diffusion equations based on distance from the tumor without inclusion of production and consumption. In particular, the moving wave of vascular growth toward the chemo attractant source is modeled as

(1) $$\frac{\partial C_{\text{stalk}}}{\partial t}=-{\mathrm{vC}}_{\mathrm{tip}}-\gamma C_{\text{stalk}}$$

(2) $$\frac{\partial C_{\mathrm{tip}}}{\partial t}=\frac{\partial \left({\mathrm{vC}}_{\mathrm{tip}}\right)}{\partial x}+\alpha C_{\text{VEGF}}C_{\mathrm{tip}}-\beta C_{\text{stalk}}C_{\mathrm{tip}}$$

(3) $$v=\chi \frac{\partial C_{\text{VEGF}}}{\partial x}$$

where $C_{\text{stalk}}$, $C_{\mathrm{tip}}$, and $C_{\text{VEGF}}$ are the concentrations of stalk cells, tip cells, and VEGF, v is the velocity of the tip cells, α is a proliferation constant, β is an anastomosis constant, γ is a decay constant, and χ is an elongation rate constant. The approach showed reasonable agreement with empirical data in growth and regression cases.

Building upon Balding and McElwain's seminal work, Anderson and Chaplain (1998) proposed a continuous field approach, representing fibronectin concentration in the tissue. They hypothesized that both chemotactic and haptotactic forces were necessary to describe endothelial cell motion toward a tumor. They tested by constructing a continuous model similar to Balding and McElwain and then discretizing that model to allow for the tracking of the movement of specific endothelial cells. Movement of tip cells was considered as being driven by three fluxes: (i) chemotaxis responding to TAF gradients; (ii) haptotaxis responding to fibronectin gradients; and (iii) a random walk term modeling unknown contributions. Thus, the vascular growth model proposed by Anderson (1998) continuous is described as

(4) $$\frac{\partial C_{\text{endo}}}{\partial t}=D_{n}\nabla^{2}C_{\text{endo}}-\nabla \cdot \left(\chi \left(C_{\text{VEGF}}\right)C_{\text{endo}}\nabla C_{\text{VEGF}}\right)-\nabla \cdot \left(p_{0}C_{\text{endo}}\nabla C_{\text{fibro}}\right)$$

(5) $$\frac{\partial C_{\text{fibro}}}{\partial t}={\mathrm{wC}}_{\text{endo}}-\mu C_{\text{endo}}C_{\text{fibro}}$$

(6) $$\frac{\partial C_{\text{VEGF}}}{\partial t}=-\lambda C_{\text{endo}}C_{\text{VEGF}}$$

where $C_{\text{endo}}$, $C_{\text{fibro}}$, and $C_{\text{VEGF}}$ are the concentrations of endothelial cells, fibronectin, and VEGF, $D_{n}$ is a cell random motility constant, χ ($C_{\text{VEGF}}$) is a chemotactic elongation rate function, $p_{0}$ is haptotactic movement rate constant, w is a fibronectin production constant, μ is a fibronectin uptake constant, and λ is a VEGF uptake constant.

In addition, given that the tip cells are supported by the stalk cells in the sprout, they assumed that the vessels would follow the path of the capillaries in a snail trail like behavior. This discretization formed the basis of an early attempt at analyzing cellular level behaviors from a tissue level perspective. Experiments with this discrete model showed that in the absence of haptotactic action, chemotaxis dominated the system and the endothelial cells experienced minimal lateral migration resulting in minimal branching and a lack of anastomosis. The introduction of haptotaxis resulted in significant lateral movement, which is in agreement with the branching and anastomosis behaviors responsible for the characteristic brush border phenomenon seen experimentally. This is evidence supporting the hypothesis that both chemotactic and haptotactic forces were necessary to predict endothelial cell motion toward a tumor. These detailed tipping cell dynamics enriched the biological description of the model, although the computational cost restricts the simulation to smaller vessel networks.

Plank and Sleeman (2004) proposed an alternative circular random walk model for endothelial cell motion. This approach relaxes the regular lattice constraint describing a more natural movement of the tip cells. In this format, chemotaxis is implemented as a turning force that seeks to pull the direction element of tip cell migration in alignment with the gradient of concentration. Rather than forming a system of PDEs that represent shifts in cell density, the movement of individual cells is tracked and assigned values for speed and direction. This extension of the model accounts for anti‐angiogenic factors, allowing a better fit of the model in terms of capillary turning rates and branching angles.

More recent works continue improving the mechanisms of tissue level models. Flegg et al. (2012) developed a model of angiogenesis in the wound healing case, which directly linked the effects of oxygen concentration to tip cell and blood vessel densities without the intermediate step of a growth factor. Zheng et al. (2013) looked into the effects of angiopoietins (Ang1 and Ang2) as well as platelet‐derived growth factor‐B on sprout initiation, extension, and vessel maturation. Connor et al. (2015) included basic fibroblast growth factor to model the effect of a dynamic extracellular environment. Pillay et al. (2017) attempt to bring the tissue level approach full circle by developing a discrete cell actor in a form which can be homogenized in the tissue level model to assess mechanisms and compare with phenomenological approaches like the stochastically average tip cells of Capasso and Morale (2009). Finally, Moreira‐Soares et al. (2018) produced a model focused on the effect of oxygen supply as driven by complete loops and its effect on the anastomosis process.

These tissue level approaches are strong at modeling the phenomenological effects of angiogenesis across tissue, however they restrict the mechanistic interpretation of biological processes at the cellular and molecular levels. Although tissue level approaches can statistically approximate such cellular behaviors, they have limited applicability in complex scenarios because the formulation inherently make assumptions about the underlying cellular level conditions.

### 3.2 Cellular level models

With advances in our understanding of the biology and the processes that drive cellular level activities, a branch of angiogenesis modeling spawned which looked at the problem specifically from a cellular level. These models have the advantage of describing the fundamental mechanisms involved in the biological processes related to vascular adaptation. This lower level description allows for the relaxation of many of the inherent assumptions of tissue level models, enabling the study of more complex scenarios and physiological conditions.

Bauer et al. (2007) explored this approach by heavily focusing on the nature of cells as individual actors using a cellular Potts model. They hypothesized that the specific local microenvironment influences the cell interactions and the resulting vessel structure in a meaningful manner during the angiogenesis process. To evaluate this, they proposed a coupled model with extracellular, cellular, and intercellular components. The extracellular component describes the diffusion and uptake of VEGF concentrations using partial differential equations as was done in the tissue level models. The cellular component uses a cellular Potts model to describe cell movement, growth and the interaction with the extracellular matrix. The intercellular component describes cell control mechanisms and signaling dependent processes. Because of the direct focus on detailed cellular interactions, the scope is highly constrained with only a single endothelial cell being considered at the starting point. This cell then navigates through an explicitly defined stroma of matrix fibers, tissue cells, and interstitial fluid following a tumor induced VEGF gradient. The cellular Potts model divides the space up into a discrete grid. In this grid, the various components compete for space with intercellular actions only occurring on the surface of the endothelial cells. Layered on these rules for interaction is an energy minimization scheme which on each update step evaluates the total energy of the system and accepts the change if it is a reduction or passes a Boltzmann probability check if it is an increase. This results in a system which naturally includes chemotactic and haptotactic forces, giving rise to realistic winding path vessel segments with endothelial cells placement, capillary width, branching, and anastomosis behaviors emerging from the defined biology instead of from predefined probability densities or relative concentrations. The tight scope of this model hinders experimental validation, as it would require information on a few cells at the very beginning of the angiogenesis process. In terms of results, this work under predicts the rate of sprout extension by a factor of 2 approximately.

Further extensions of this approach moved into 3D formulations (Shirinifard et al., 2009) while others focused on exploring the effects of specific signaling pathways, like Bentley et al. (2008), who developed a model looking specifically at the notch mediated tip cell selection process at the point of sprout initialization from a capillary.

In a more recent work, Stepanova et al. (2021) present a cellular level model, which is focused on the VEGF‐Delta‐Notch signaling pathway which controls the phenotype expression of endothelial cells in the sprout. Managing endothelial cell expression, as either stalk cells or tip cells, on biological principles allows for specific implementation of mechanisms of behavioral action like branching, overtaking, and interactions with the extracellular matrix. The advantage of this approach is that characteristic behaviors like chemotactic sensitivity and elongation rate shifts emerge from the properties of the model rather than being implemented as specific rules. This allows for more realistic simulations of treatment options which target the specific implemented mechanisms like the Delta‐Notch pathway. This model was validated with in‐vitro experiments and reproduced general traits of SA such as branching, the brush border, and VEGF concentration dependent sprout elongation rates.

Cellular level models are good for examining the effect of specific pathways and responses but are limited in that their accuracy only extends to the specific interactions that are included in the model. Although these detailed discrete approaches carry less inherent assumptions about the biological systems they suffer from a limitation of scale due to computational cost considerations. In this article, we have introduced cellular models which look at the response of single cell, scaling this kind of approach to a tissue level problem is impractical as every added piece of detail increases the computational cost of the model.

### 3.3 Multilevel models: coupled tissue and cellular levels

Cellular level modeling and tissue level modeling approaches are not necessarily exclusive. There are parts of the angiogenesis problem for which the tissue level assumptions are strong, like in the case of diffusible products like oxygen and VEGF. By adopting a multilevel approach, where cellular level models are coupled with tissue level systems, it is possible to compromise between the statistical assumptions of tissue models and the computational burden intrinsic of cellular models.

Much as Anderson and Chaplain (1998) extended on Balding and McElwain (1985) by refining the tip cell dynamics, it is possible to go further by treating the cells as individual actors that follow set of rules coupled with a background continuum media that contains tissue level information. As part of this, these models tend to use complex functions to define specific interactions rather than using full PDE descriptions.

Owen et al. (2009) takes a more network focused approach to the angiogenesis problem by acknowledging that it is a multi‐process problem where the homeostasis arises as the interplay between angiogenesis and pruning. Specifically, angiogenesis processes generate vessels to satisfy the metabolic demand of the tissues, while pruning processes remove vessels due to an inability to sustain sufficient blood flow. They hypothesized that both angiogenesis and pruning behaviors were necessary to predict the topological traits of the resulting vascular networks. The cellular level model incorporates resource consumption, VEGF production, migration, multiplication, apoptosis, and tumor interactions. This is coupled with a diffusible layer containing information on the levels of VEGF and oxygen in the system. The diffusible layer is further coupled with a vascular adaptation and pruning schema which controls structural changes in vessel radii and hematocrit in response to prolonged periods of low wall shear stress. The combination of these processes allow for a vessel remodeling process that favors the formation of networks which satisfy the oxygenation requirements while minimizing unnecessary and inefficient vessel segments.

Secomb et al. (2013) extend Owen et al. by focusing further on the homeostatic balance between the opposing forces of angiogenesis and pruning. They developed a model, which uses an over‐abundant random angiogenesis process opposed by a redundant vessel pruning process to develop vessel networks capable of adapting to shifting tissue conditions. They hypothesize that structural adaptation measures are key to a vessel network's ability to respond to changing conditions. Secomb et al. describe their network as a minimal set of mechanisms that is sufficient to solve the vascular filling problem. The mechanisms they include are: (i) the generation of diffusible growth factor in hypoxic tissue; (ii) sprout formation coupled with high growth factor concentration; (iii) sprout maintenance and anastomosis; (iv) vessel diameter and blood flow adaption; and (v) a redundant pruning mechanism. The time scale of the adaptation response is longer than growth behavior which leads to rapid growth and satisfaction of oxygen needs followed by a period of optimization which removes unnecessary excess vessels. Secomb et al. compare their simulated network with an experimentally observed network. They found that the simulated network had a lower vascular density but a matching mean tissue oxygen partial pressure with a narrower distribution meaning less hypoxic tissue overall.

Liu et al. developed a model for angiogenesis in skeletal muscle, which integrated modules for microvascular blood flow, oxygen transport, VEGF ligand‐receptor interactions and transport, and a cell module describing capillary sprout formation. They explored the unique VEGF changes that result from temporary hypoxia due to increased cellular oxygen consumption (Liu et al., 2011).

Jain and Jackson (2013) developed a hybrid model based on the theory of reinforced random walk where the chemotactic effects of growth factor were achieved through the binding of angiogenic factors to individual cells rather than approximating this effect through a concentration field.

The multilevel models have also served as the platform for extending modeling approaches into 3D with works like Milde et al. (2008) and Bookholt et al. (2016) reconstructing 3D in vitro assay. The latter used a finite element mesh in which endothelial cells moved through a nonhomogeneous lattice to mimic the angiogenesis process.

Due to the multilevel nature of hybrid approaches, they naturally tend to be more comprehensive in their inclusion of a variety of contributing factors. However, due to the requirements introduced by coupling different scales together each of the components of the model have a tendency to be simpler and less detailed than models which focus on a single level.

### 3.4 Nonmechanistic computational methods

Adjacent to the modeling approaches we have discussed is a family of models that generate microvascular networks without modeling the underlying physiological mechanisms. These methods are based on energy optimality principles (Blanco et al., 2013; Jaquet et al., 2018; Karch et al., 1999, 2000; Linninger et al., 2019; Schreiner et al., 1996, 2006; Talou et al., 2021), space‐filling techniques (Lorthois & Cassot, 2010; Smith et al., 2019), and approximation via fractal patterning (Gottlieb, 1990; Zamir, 2001) to create plausible arrangements that match the functional response of microvascular networks (e.g., yields a known pressure drop or vascular resistance). As a strength, these approaches are capable of replicating certain functional responses of real networks. Their main weakness is that they are not suitable for studying the effect of physiological disruptions because these methodologies do not model the underlying biological mechanisms. Because of this, we mention their existence for completeness, but will not go into further depth about these approaches.

### 3.5 Comparative analysis

As with many other modeling efforts, the presented works increasingly added more biological mechanisms to improve the description of microvascular development and adaptation (see Table 1. At the tissue level, vascular development in tumoral growth was initially modeled by only accounting by growth factor‐driven angiogenesis (Balding & McElwain, 1985). The addition of mechanisms for tip cells‐tissue interactions served to improve the description of cellular kinematics yielding more accurate vascular networks (Anderson & Chaplain, 1998; Plank & Sleeman, 2004). To continue to refine the description of cellular kinematics required the development of cellular level models yielding to the opportunity to describe the mechanisms of interaction between tip cells, growth factors, and the extracellular matrix (Bauer et al., 2007). The later work demonstrated that accounting for cellular mechanisms is key to naturally describe the topological and geometric complexities intrinsic to SA. Most of the following works building on mechanistic biological complexity presented a common trend of adopting multilevel models to couple cellular and tissue mechanisms. The next significant increase in complexity was the modeling of hemodynamics to compute oxygen distribution and wall shear stress fundamental drivers for VEGF production, arteriogenesis, and pruning Owen et al., 2009; Secomb et al., 2013).

**TABLE 1:** Comparative analysis of biological processes, driving forces, and organization levels modeled in the referenced computational models for microvascular development and remodeling

	Biological processes					Driving factors					
Referenced work	SA	IA	An	Ar	P	$O_{2}$	MR	GF	Inh	WSS	Organization level
(Balding & McElwain, 1985)	✓							‐			Tissue
(Anderson & Chaplain, 1998)	✓		✓					‐			Tissue
(Plank & Sleeman, 2004)	✓		✓					‐			Tissue
(Bauer et al., 2007)	✓		✓					✓			Cellular
Owen et al., 2009)	✓		✓	✓	✓	✓	‐	✓		✓	Multilevel
(Secomb et al., 2013)	✓		✓	✓	✓	✓	‐	✓		✓	Multilevel
(Pillay et al., 2017)	✓		✓					‐			Multilevel

Abbreviations: ✓, modeled; ‐, fixed or partially modeled; An, anastomosis; Ar, arteriogenesis; GF, growth factors; IA, intussusceptive angiogenesis; Inh, inhibitors; MR, metabolic requirements; $O_{2}$, oxygenation; P, pruning; SA, sprouting angiogenesis; WWS, mechanical stresses.

The comparison between the main works in the area (see Table 1) clearly shows that IA has been largely overlooked. This is in part caused by the lack of understanding in the IA signaling pathway and triggering mechanisms hindering the proper modeling of such process. Nonetheless, as we described in Section 2.1.3, IA could be the main driving process in later stages of vascular development accounting for the genesis of most of the vessels in the cardiovascular system. Lastly, inhibition factors and metabolic requirements other than oxygen, also present some opportunities for future research and the development of more accurate microvascular development and adaptation models.

## 4 APPLICATIONS

The computational models discussed in the previous sections allow us to explore our understanding of the mechanisms involved in microvascular development and adaptation. Hence, it provides a framework to make predictions of the revascularization processes when environmental conditions are altered. Due to its in silico nature, the history of these developmental and adaptation processes are fully described by the models informing the causation and individual mechanisms within the process. This detailed understanding brings a plethora of opportunities for research and clinical applications. As follows, we will enumerate a few interesting opportunities unlocked by these computational tools.

### 4.1 Homeostasis and adaptation of vasculature

In normal healthy individuals, angiogenesis occurs throughout the growth process and homeostatically as part of the continuous upkeep of the body. Of key interest are the areas of ocular formation and muscular development. The eye is interesting because of the unique formation and subsequent reabsorption of a vascular network to create the avascular elements of the eye without the vessels remaining to obscure vision (Gariano & Gardner, 2005). Computational modeling of this problem allows exploration of the mechanics of a delicate system while minimizing the need for animal‐based experimentation.

The muscles are interesting because of the variation in demand and supply characteristics with changes in the level of exercise (Hoier & Hellsten, 2014) with capillary density shifting to match the increased blood flow demands of high intensity exercise. Angiogenesis is also key to the muscular pathologies related to peripheral arterial disease (Kikuchi et al., 2014) and diabetes (Kivelä et al., 2006). Mathematical modeling of the muscles has applications in athletic optimization and isolating the factors contributing to muscle growth.

### 4.2 Modeling tumoral growth

Pathologically uncontrolled angiogenesis is one of the key contributors to the growth of cancerous tumors. Most of the models analyzed in this article approached the problem from the tumor perspective. In the tumor case, angiogenic growth factors are released in excess quantities (Hicklin & Ellis, 2005), it is through this mechanism that tumors gain access to the resources necessary to achieve uncontrolled growth (Kerbel, 2008).

Theoretically, controlling angiogenesis would allow for the slowing of tumor development and a targeted angiogenic antagonist could be used to kill tumors by cutting off their supply of resources (Weis & Cheresh, 2011). Angiogenesis modeling contributes to the realism of in silico assays of the tumor problem which can be used for exploration of specific treatment options and extrapolation of growth profiles (Rieger & Welter, 2015).

### 4.3 Non‐tumor pathologies

Applications exist in pathology beyond the tumor case. Specifically cases where a significant disruption has occurred to the existing vascular network and angiogenesis is needed to form a new vascular network. In wound healing the clotted tissue is invaded by angiogenic sprouts forming a vascular network, as healing proceeds this network recedes to homeostatic levels (Flegg et al., 2015; Tonnesen et al., 2000).

In the case of blood clots and strokes, a vessel has become occluded so as to prevent blood flow leading to tissue degeneration downstream of the occlusion. Revascularization by way of angiogenesis is key to recovery following an ischaemic event (Ergul et al., 2012; Ruan et al., 2015). Mathematical modeling allows for predictions about the performance of post recovery tissues.

### 4.4 Organs printing

Turning to the future, organ printing and the fabrication of artificial tissue requires the support of a full vascular network to ensure supply just as in natural tissue (Visconti et al., 2010). Angiogenesis is the process of growth for the smallest scale of the necessary vascular network and is thus central to the development of these supply systems.

## 5 CONCLUSION

In this article, we have assembled an overview of the biological background behind the revascularization processes, and the computational models that have been developed to emulate such processes in silico. We have identified that the models fall into two broad categories; continuum modeling approaches that phenomenologically track the concentrations of the different components of angiogenesis, and discrete cell actor approaches which model the behaviors of single cells. Although most of the methods we highlighted focused on tumoral growth, the models developed can be more widely adapted for use in a variety of applications.

We found that core focus of the models presented was on the sprouting process and how tip cells interacted with their environment. We have presented a variety of implementations for the chemotactic and haptotactic responses of tip cells. Comparatively little focus has been placed on the complimentary processes of intussusceptive angiogenesis and pruning. In relation to the driving factors of vascular adaptation, the focus has been on growth factors and oxygen concentrations with other metabolites and inhibitory elements remaining mostly unexplored.

Tissue level, cellular level, and multilevel models all constitute valid ways of modeling the revascularization processes with different strengths and weaknesses. In choosing what kind of modeling approach to take the most important consideration is suitability to the application. For instance in the muscle application, hemodynamics are a key component and, as such, tissue level or multilevel models are suitable. Conversely, applications looking at the effect of a single signaling hormone on endothelial cell expression are more appropriately modeled by a cellular level model. The focus on tumoral growth as the primary application of most of the reviewed models is likely responsible for the focus on sprouting and growth factor interactions as the dominance of these components are core to the pathology.

This review does not constitute an exhaustive list of all approaches that have been taken in modeling the development and adaptation of microvascular networks or the full extent of the biological understanding. Rather the aim of this article is to provide a review of the core approaches in the area with a focus on examining the computational models through the lens of the underlying biological processes.

## AUTHOR CONTRIBUTIONS

**Cameron Apeldoorn:** Conceptualization (supporting); formal analysis (equal); investigation (lead); methodology (equal); writing – original draft (lead); writing – review and editing (lead). **Soroush Safaei:** Formal analysis (supporting); methodology (supporting); supervision (supporting); writing – review and editing (equal). **Julian Paton:** Conceptualization (supporting); formal analysis (supporting); supervision (supporting); writing – review and editing (equal). **Gonzalo Maso Talou:** Conceptualization (lead); formal analysis (equal); investigation (supporting); methodology (equal); supervision (lead); writing – original draft (supporting); writing – review and editing (lead).

## FUNDING INFORMATION

Cameron Apeldoorn and Gonzalo D. Maso Talou gratefully acknowledge The University of Auckland Foundation for the support in this research and The University of Auckland for Cameron Apeldoorn's doctoral scholarship. Soroush Safaei acknowledges the support of the Aotearoa Foundation.

## CONFLICT OF INTEREST

The authors declare no conflict of interest.

## RELATED WIREs ARTICLES

Integrative models of vascular remodeling during tumor growth

## Data Availability

Data sharing is not applicable to this article as no new data were created or analyzed in this study.
